# *Thymus musilii* Velen. Methanolic Extract: In Vitro and In Silico Screening of Its Antimicrobial, Antioxidant, Anti-Quorum Sensing, Antibiofilm, and Anticancer Activities

**DOI:** 10.3390/life13010062

**Published:** 2022-12-25

**Authors:** Emira Noumi, Iqrar Ahmad, Nouha Bouali, Harun Patel, Siwar Ghannay, Ayshah Aysh ALrashidi, Mohammad A. Abdulhakeem, Mitesh Patel, Ozgur Ceylan, Riadh Badraoui, Afnan Elayyan Mousa Elayyan, Mohd Adnan, Adel Kadri, Mejdi Snoussi

**Affiliations:** 1 Department of Biology, College of Science, University of Ha’il, P.O. Box 2440, Hail 81451, Saudi Arabia; 2 Laboratory of Genetics, Biodiversity and Valorization of Bio-Resources (LR11ES41), Higher Institute of Biotechnology of Monastir, University of Monastir, Avenue Tahar Haddad, BP74, Monastir 5000, Tunisia; 3 Department of Pharmaceutical Chemistry, Prof. Ravindra Nikam College of Pharmacy, Gondur, Dhule 424002, India; 4 Division of Computer Aided Drug Design, Department of Pharmaceutical Chemistry, R. C. Patel Institute of Pharmaceutical Education and Research, Shirpur 425405, India; 5 Department of Chemistry, College of Science, Qassim University, P.O. Box 6688, Buraidah 51452, Saudi Arabia; 6 Centre of Research for Development, Department of Biotechnology, Parul Institute of Applied Sciences, Parul University, Vadodara 391760, India; 7 Ula Ali Kocman Vocational School, Mugla Sitki Kocman University, Mugla 48147, Turkey; 8 Department of Histo Embryology and Cytogenetics, Medicine Faculty of Sfax, University of Sfax, Road of Majida Boulia, Sfax 3029, Tunisia; 9 Department of Clinical Laboratory Science, College of Applied Sciences-Qurayyat, Jouf University, P.O. Box 2014, Sakaka 72388, Saudi Arabia; 10 Faculty of Science and Arts in Baljurashi, Albaha University, P.O. Box 1988, Albaha 65527, Saudi Arabia; 11 Department of Chemistry, Faculty of Science of Sfax, University of Sfax, B.P. 1171, Sfax 3000, Tunisia

**Keywords:** *Thymus musilii* Velen., methanolic extract, antimicrobial, antioxidant, anti-quorum sensing, ADME, molecular docking

## Abstract

*Thymus musilii* Velen. is a rare plant species cultivated in the Ha’il region (Saudi Arabia) under greenhouse conditions. In this work, we described, for the first time, the phytochemical composition, antimicrobial, antioxidant, anti-quorum sensing, and anticancer activities of *T. musilii* methanolic extract using both experimental and computational approaches. The obtained results showed the identification of eight small-like peptides and eighteen phyto-compounds by using high-resolution liquid chromatography–mass spectrometry (HR-LCMS) dominated mainly by compounds belonging to isoprenoid, fatty acyl, flavonoid, and alkaloid classes. The tested extracts exhibited high antifungal and antibacterial activity with the mean diameter of growth inhibition zones ranging from 12.33 ± 0.57 mm (*Pseudomonas aeruginosa* ATCC 27853) to 29.33 ± 1.15 mm (*Candida albicans* ATCC 10231). Low minimal inhibitory concentrations were recorded for the tested micro-organisms ranging from 0.781 mg/mL to 12.5 mg/mL. While higher doses were necessary to completely kill all tested bacterial and fungal strains. Thyme extract was able to scavenge DPPH^•^, ABTS^•+^, β-carotene, and FRAP free radicals, and the IC_50_ values were 0.077 ± 0.0015 mg/mL, 0.040 ± 0.011 mg/mL, 0.287 ± 0.012 mg/mL, and 0.106 ± 0.007 mg/mL, respectively. The highest percentage of swarming and swimming inhibition was recorded at 100 µg/mL with 39.73 ± 1.5% and 25.18 ± 1%, respectively. The highest percentage of biofilm inhibition was recorded at 10 mg/mL for *S. typhimurium* ATCC 14028 (53.96 ± 4.21%) and *L. monocytogenes* ATCC 7644 (49.54 ± 4.5 mg/mL). The in silico docking study revealed that the observed antimicrobial, antioxidant, and anticancer activities of the constituent compounds of *T. musilii* are thermodynamically feasible, notably, such as those of the tripeptides (Asn-Met-His, His-Cys-Asn, and Phe-His-Gln), isoprenoids (10-Hydroxyloganin), and diterpene glycosides (4-Ketoretinoic acid glucuronide).

## 1. Introduction

The global rise in infectious diseases due to antibiotic-resistant strains of bacteria is becoming a global health problem, and, in turn, potentially profound and complicated ailments, such as diabetes, cardiovascular diseases, and cancer, lead to significant causes of death and morbidity worldwide [1,2,3]. The growing resistance of microbes against commercial drugs is one of the main reasons of concern for both researchers and clinicians. Besides that, the side effects in the host caused by antibiotics, especially when increasing the dosage of the drug to suppress infections, induce allergic reactions, diarrhea, nausea, and drug–drug interactions [4,5,6]. Thus, to encompass the ongoing spread of antimicrobial resistance as well as the excessive and unregulated use of antibiotics, scientists have been motivated to search for new agents and alternative antibiotics to combat bacterial pathogens. One of the most ideal approaches to fight bacterial infections is to eliminate virulence factors that are often involved in pathogenicity and infections [7,8].

The genus *Thymus* belongs to the *Lamiaceae* family and is very popular due to its large use as a remedy in folk medicine and as a condiment, mainly in the Mediterranean zone. The Kingdom of Saudi Arabia has counted at least three species of *Thymus*, namely, *T. bovei* Benth., *T. decussatus* Benth., and *T. musilii* Velen. Additionally, *T. vulgaris* is largely cultivated in many provinces of the kingdom. In this study, we focused on the *T. musilii* Velen. species, which is largely distributed in Iraq, Palestine, and Saudi Arabia [9]. Traditionally, local Saudi Arabians used the leaves and flowers of this plant as a garnish, added it to a variety of foods or for preparing infusion tea, as well as to cure several microbial diseases. Thyme is widely cultivated in Mediterranean countries, where it is exported to several countries across the world, whether as dried leaves or in the form of volatile oils. Despite the economic and environmental importance of this plant, it is considered one of the endangered plants of several environments in the Ha’il region.

Medicinal plants are a rich reservoir of bioactive molecules, having specific biochemical and organoleptic characteristics with an imminent potential to promote health and to be explored as promising therapeutic drugs [10,11,12,13,14,15,16]. In fact, it was previously demonstrated that *T. musilii* essential oil is a rich source of oxygenated monoterpenes (87.010 ± 0.279%), monoterpene hydrocarbons (11.013 ± 0.039%), and sesquiterpene hydrocarbons (1.953 ± 0.005%). The same authors reported that the same *T. musilii* essential oil was dominated by thymol (67.697 ± 0.938%), thymyl acetate (12.933 ± 0.221 %), and ***p***-cymene (4.617 ± 0.0.119 %) [16]. Bioactive plant-based products have been recognized as a useful resource that may lead to the discovery of new antimicrobial substances with possibly new mechanisms of action. Extensive work has been conducted exploring the effect of various extracts and essential oils of plants in traditional medicine, with a great interest in these compounds as alternative remedies to cure various ailments [16,17]. Additionally, infectious diseases are responsible for many diseases in humans, including cardiovascular and neurodegenerative diseases due to an imbalance of redox states, involving either the excessive generation of reactive oxygen species (ROS) or the dysfunction of the antioxidant system [18,19].

Traditionally, in natural chemistry fields, researchers used to isolate and characterize chemical constituents and evaluate them for their biological activities. Molecular docking has become an increasingly important tool for computed drug discovery [20]. The docking method could be used to simulate the interaction between a small molecule and a protein at the atomic level, allowing us to assess small molecule behavior in target protein binding sites, as well as explain fundamental biochemical processes [21,22]. The biological effect of a plant extract is recognized in phytochemical screening, but it cannot indicate which phyto-constituent exerts an action. As a result, in silico docking studies are essential for comprehending the interaction and affinity of identified phytochemicals with biological targets [16,22,23,24]. Hence, it could be deduced that in silico analyses usually mirror or confirm the in vitro and in vivo findings and confirm the biological effects of the assessed compounds [25].

Hence, the aim of this study was to investigate, for the first time, the chemical composition of organic extracts from *T. musilii* Velen. cultivated in the Ha’il region by using the HR-LCMS technique to study its antioxidant activities. The ability of *T. musilii* plant extracts to kill pathogenic micro-organisms using disc diffusion and microdilution assays and to attenuate the secretion of some *Pseudomonas aeruginosa* virulence-related properties controlled by the quorum sensing system were also studied. The effect on colon, breast, and lung cancer cells were also investigated. Moreover, ADME profiles and molecular docking targeting selected proteins were also analyzed.

## 2. Materials and Methods

### 2.1. Plant Material Sampling

The plants used in this study were collected in October 2019 from a nursery belonging to the Ministry of Agriculture in the region of Ha’il (Al-Gaad, Ha’il, Saudi Arabia). A voucher specimen (AN 001) was deposited in the Department of Biology, at the University of Ha’il, Saudi Arabia [16]. Methanolic extract was performed by using the protocol previously described by Haddaji et al. [23]. For the experiment, the powdered flowering aerial parts of *T. musilii* (4 g) were mixed with pure methanol (40 mL) in a dark glass bottle with agitation for three days at room temperature. Then, the crude extract was filtrated by using Whatman filter paper (No. 1). The methanolic extract was then removed using a rotary evaporator (BUCHI Labortechnik GA, CH-9230 Flawil 1, Switzerland). Finally, the concentrated extract was stored at 4 °C until the actual experiment was conducted. The yield of extraction was calculated after three running cycles and expressed according to the dry weight.

### 2.2. Phytochemical Screening

#### 2.2.1. Quantification of Total Phenols, Total Flavonoids, and Total Condensed Tannins

Total phenol content (TPC) was estimated according to the Folin–Ciocalteu method, as described by Kumar et al. [26]. An aliquot of 100 μL from *T. musilii* stock solution (1 mg/mL) was added to 750 μL of Folin–Ciocalteu reagent (Sigma Aldrich, Germany). The liquid mixture was allowed to stand for 5 min before 750 μL of saturated Na_2_CO_3_ solution was added. After 90 min of incubation in the shade and at room temperature, absorbance was recorded at 725 nm using a UV-Vis spectrophotometer (Agilent Cary 60 UV-Vis, USA). The obtained data were expressed as milligram (mg) of gallic acid equivalents per gram of plant extract (mg EGA/g extract). Ten milligrams of gallic acid were dissolved in methanol-50% (100 μg/mL) and then further diluted to 0.78, 1.56, 3.125, 6.25, 12.5, or 25 μg/mL. The quantification was completed based on the standard calibration extinction curve of gallic acid concentrations (y = 6.9326 x − 0.0141; R^2^ = 0.9981).

Total flavonoid content (TFC) was determined using the Aluminum chloride (AlCl_3_) method developed by Benariba et al. [27]. For the experiment, 1.5 mL of *T. musilii* extract (1 mg/mL) was mixed with 1.5 mL of AlCl_3_-6H_2_O solution (2%). The mixture was vortexed gently and incubated for 10 min at room temperature. Absorbance was recorded at 367 nm against the blank (the same mixture without the sample). Total flavonoid amounts were expressed as milligram (mg) of quercetin equivalents per gram of plant extract (mg QE/g Ext) for each organ. The standard calibration curve of quercetin was conducted (dilutions about 1.56, 3.125, 6.25, 12.5, or 25 μg/mL). Quantification was completed based on quercetin standard curve (y = 12.234 x − 0.0894; R^2^ = 0.9997).

Total tannin content (TTC) was determined by a colorimetric method using modified vanillin assay [28]. Then, 3 mL of 4% methanolic vanillin solution and 1.5 mL of concentrated H_2_SO_4_ were added to 50 µL of extract (1 mg/mL). The mixture was allowed to stand for 15 min, and the absorbance was measured at 500 nm against methanol or water as blank. Total condensed tannin concentration was expressed as mg catechin/g dry weight (mg CE/mg). All samples were analyzed in three replications. The standard calibration curve of catechin was conducted (dilutions about 0.039, 0.78, 1.56, 3.125, 6.25, 12.5, or 25 μg/mL). Quantification was completed based on quercetin standard curve (y = 14.107 x + 0.0079; R^2^ = 0.9987).

#### 2.2.2. Identification of Bioactive Compounds by HR-LCMS Technique

The methanolic extract from *T. musilii* aerial parts was studied for the presence of various phytochemical compounds by using HPLC-DAD-ESI/MSn technique, following the method described by Noumi et al. [24].

### 2.3. Screening of the Biological Activities

#### 2.3.1. Antimicrobial Activities

The antimicrobial activities of *T. musilii* methanolic extract was screened against *Escherichia coli* ATCC 35,218 (*E. coli*), *Pseudomonas aeruginosa* ATCC 27853 (*P. aeruginosa*), *Proteus mirabilis* ATCC 29245 (*P. mirabilis*), *Klebsiella pneumoniae* ATCC 27736 (*K. pneumoniae*), two multidrug-resistant *Staphylococcus aureus* (*S. aureus*) strains, and *Enterobacter cloacae* (*E. cloacae*). In addition, fungal strains were also tested (*C. albicans* ATCC 10231, *Cryptococcus neoformans* ATCC 14116, *C. vaginalis* (clinical strain), *Candida* sp. (clinical strain), *Aspergillus fumigatus* ATCC 204,305, and *Aspergillus niger*. Disc diffusion assay was used (20 µL/disc) using the same protocol described by Snoussi et al. [29] for the determination of the diameter of growth inhibition zone estimated on agar medium. The optical density of all bacterial suspensions was adjusted, and bacterial cell density was about 1 × 10^7^ UFC/ml (OD_600_ = 0.1). While the cell density of the tested yeast strains diluted in glass tubes containing sterile saline (0.8% NaCl) was adjusted to 1 × 10^7^ UFC/ml (OD_540_ = 0.5) [30]. Using a sterile cotton swab, each tested micro-organism was spread on the surface of agar media (Mueller–Hinton for bacteria and Sabouraud Chloramphenicol agar for fungi). Sterile 6 mm discs (Biolife, Milan, Italy) were impregnated with 10 µL/disc from a stock solution of methanolic extract (200 mg/mL) and deposited in triplicate on the surface of each inoculated plate. All Petri dishes were incubated overnight at 37 °C, and the mean diameter of growth inhibition zone was recorded and expressed as mGIZ ± SD (mm).

To estimate the minimal inhibitory concentrations (MIC values expressed in mg/mL) and minimal bactericidal/fungicidal concentrations (MBC and MFC values), the obtained extract was serially diluted in DMSO-5% supplemented with 0.025% tween 80 (from 100 mg/mL to 0.097 mg/mL) in 96-well microtiter plates containing 95 µL of the microbial suspension and 95 µL of the enrichment broth (Lauria–Bertani for bacteria and Sabouraud dextrose broth for fungal strains). MIC concentration is estimated visually and is defined as the lowest concentration needed to inhibit the growth of each tested micro-organism in the wells. While MBC and MFC concentrations are defined as the lowest concentration needed to kill 99.99% of the tested micro-organisms and are confirmed by point-inoculating MH and SC media from wells with concentrations higher than the recorded MIC value for each micro-organism tested [30].

#### 2.3.2. Antioxidant Activities

The ability of *T. musilii* extracts to scavenge the DPPH^•^ stable free radicals was determined following the same method as previously reported by Mseddi et al. [16]. The method of Koleva et al. [31] was used for β-Carotene bleaching test and ferric reducing antioxidant power [32], and the Oyaizu method for the determination of reducing power was used [33]. The radical-scavenging activity of the extracts against ABTS + (2,2′-azino-bis(3-ethylbenzothiazoline-6-sulfonic acid)) radical cations was measured using the same protocol described by Hamdi et al. [28].

##### DPPH Radical-Scavenging Activity Assay

For the experiment, a stock solution (20 mg/mL) from *T. musilii* extract and the standard (stock solution 1 mg/ml) at different concentrations were pipetted in separate test tubes. A volume of 0.5 mL of each sample and standard was mixed with the same volume of DPPH• methanolic solution. The mixture was shaken vigorously and allowed to stand for 30 min in darkness and at a temperature of 25 °C. The absorbance of the resulting solution was measured at 520 nm with a spectrophotometer. All measurements were performed in triplicate. A mixture of 0.5 mL of DPPH• solution and 0.5 mL of methanol were taken as a control. Pure methanol was taken as a blank. Inhibition of free radical DPPH• as a percentage (PI%) was calculated by the following equation (Equation (1)):

PI% = [(A_Control_ − A_Sample_)/A_Control_] × 100 (1)

where A_Control_ and A_Sample_ are the absorbances of the control solution and of a test sample or standard, respectively. The IC_50_ corresponding to the concentration needed to scavenge 50% of DPPH• free radicals were estimated from the dose–response curve by using Graph-Pad 8 software (San Diego, CA, 92108-2711 USA).

##### ABTS Radical-Scavenging Activity Assay

The antiradical assay was performed using 2,2′-azino-bis (3-ethylbenzthiazoline-6-sulphonic acid), commonly called the ABTS cation scavenging activity test. The radical monocation of ABTS was generated by reacting ABTS solution (7 mM) with 2.45 mM K_2_S_2_O_8_. The mixture was allowed to stand for 15 h in the dark at room temperature. Thyme extract was dissolved in methanol for the organic extracts and distilled water for the aqueous extract. The different concentrations of *T. musilii* extract and of the tocopherol (vitamin E) standard were tested. The standard was used for comparison. The measure of the antioxidant activity was realized by adding 200 µL of each standard and sample to 800 µL of diluted ABTS·+. The absorbance was measured spectrophotometrically at 734 nm after 30 min. All measurements were performed in triplicate. The antioxidant capacity of the test sample and standard were expressed as percent of inhibition (%). The percentage of scavenging of ABTS. + was calculated by Equation (1). The IC_50_ corresponding to the concentration needed to scavenge 50% of ABTS. + radicals were estimated from the dose–response curve by using Graph-Pad Prism software.

##### Β-Carotene/Linoleic Acid Method

The formation of a free radical from linoleic acid was obtained after heating the β-carotene/linoleic acid complex. A total of 2 mL of β-carotene solution (1.5 mg of β-carotene/2.5 mL of chloroform) was added to 20 μL of linoleic acid, and 200 μL tween 20 was mixed. The chloroform was removed at 40 °C under vacuum using a rotary evaporator. A volume of 50 mL of distilled water was added to the dried mixture to form a β-carotene–linoleic acid emulsion. To determine the β-carotene bleaching activity of each extract, 0.800 mL of the emulsion was added to 0.200 mL of *T. musilii* extract at different concentrations (stock solution 20 mg/mL) and the standard (stock solution 1 mg/ml). The mixtures were incubated in a water bath at 50 °C for 120 min, and the absorbance was estimated at 470 nm before and after incubation. Tests were carried out in triplicate. The antioxidant activity of extracts was calculated using the following equation (Equation (2)):

PI% = [1 − (A_0_ − A_t_/A_c0_ − A_ct_)] × 100 (2)

where A_0_ and A_c0_ refer to the absorbance values measured at zero time for the test sample or standard and the control, respectively, and At and Act refer to the corresponding absorbance values of the test sample or standard and the control measured after incubation for 120 min, respectively. The IC_50_ corresponding to the concentration needed for β-carotene bleaching inhibition at 50% was estimated from the dose–response curve by using Graph-Pad Prism software.

##### Reducing Power

The reduction of Fe^3+^ to Fe^2+^ was determined by measuring absorbance of the Perls Prussian blue complex. This method is based on the reduction of (Fe^3+^) ferricyanide in stoichiometric excess relative to the antioxidants. For this purpose, different concentrations (0.0312, 0.0625, 0.125, 0.25, 0.5, and 1 mg/mL) of *T. musilii* extract were mixed with 1 mL of 0.2 M sodium phosphate buffer (pH 6.6) and 1 mL (1%) of potassium ferricyanide [K_3_Fe(CN)_6_]. The mixture was incubated at 50 °C for 20 min and then acidified with 1 mL of trichloroacetic acid (10%). Finally, 0.25 mL of FeCl_3_ (0.1%) was added to this solution. Distilled water was used as blank and for control. Absorbance of this mixture was measured at 700 nm using a UV spectrophotometer. Increased absorbance indicates ferric reducing power capability of the sample. The IC_50_ corresponding to the concentration needed for reduction of Fe^3+^ to Fe^2+^ at 50% was estimated from the dose–response curve by using Graph-Pad Prism software.

#### 2.3.3. Effect on *Pseudomonas aeruginosa* PAO1 Motility

*P. aeruginosa* PAO1 starter strain was used to study the effect of *T. musilii* methanolic extract on its flagellar motility (swarming and swimming) using the same procedure described by Snoussi et al. [34]. Three different concentrations of *T. musilii* methanolic extracts were used (50, 75, and 100 µg/mL). All experiments were conducted in triplicate.

#### 2.3.4. Effect on Biofilm Formation by Pathogenic Bacteria and Yeast

The effect of *T. musilii* methanolic extract to inhibit the biofilm formation on 96-well plates by *S. aureus* ATCC 25923, *E. faecalis* ATCC 19433, *E. coli* ATCC 25922, *P. aeruginosa* ATCC 27853, *Salmonella typhimurium* ATCC 14028, *L. monocytogenes* ATCC 7644, and *C. albicans* ATCC 10,239 was tested by crystal violet technique [35]. For the experiment, 50 µL of an overnight-grown micro-organism culture and tryptone broth was prepared at different concentrations of *T. musilii* extracts (1, 1/2, 1/4, 1/8, and 1/16 MIC concentration value). The percentage of biofilm inhibition (expressed in percentage) was calculated as previously described by Ceylan et al. [36].

### 2.4. Anticancer Activities

*T. musilii* methanolic extract was tested against human lung (A549), breast (MCF-7), and colon (HCT-116) cancer cells. For the experiment, cell lines were cultured upon 80% confluence, and 0.4% Trypan Blue solution (Hi-Media, India) (0.4%) was used to stain the cells. Then, cells were treated for 48 h with various *T. musilii* concentrations ranging from 100 μg/mL to 500 μg/mL. Afterward, 200 μL of medium containing 10% MTT reagent was added to each well to obtain a final concentration of 0.5 mg/mL, and the plates were incubated for a further 3 h at 37 °C with 5% CO_2_ atmosphere. After removing the medium, 100 μL of DMSO was added to dissolve the formed formazan crystals. The absorbance was measured at 570 nm and 630 nm. The percentage growth inhibition was calculated after subtracting the background and the blank, and the concentration of the test drug needed to inhibit cell growth by 50% (IC_50_) was calculated from the dose–response curve for the respective cell line [37,38].

### 2.5. In Silico Approach

#### 2.5.1. ADME Study

The pharmacokinetic, drug-likeness, and medicinal properties of the identified molecules from *T. musilii* methanolic extract were studied using free online software (SwissADME: http://www.swissadme.ch/; accessed on 2 October 2022) [39]. 

#### 2.5.2. Molecular Docking Study

A molecular docking study was undertaken to elucidate the binding affinity and molecular basis of the interaction of identified compounds. The antibacterial, antifungal, and antioxidant properties of the macromolecules were tested against *S. aureus* TyrRS (PDB ID: 1JIJ), *S. aureus* DNA gyrase (PDB ID: 2XCT), human peroxiredoxin 5 (PDB ID: 1HD2), *C. albicans* Sap 1 (PDB ID: 2QZW), MLK4 kinase domain (PDB ID: 4UYA), human kinesin (PDB ID: 4BBG), and BRCT protein (PDB ID: 1JNX) key enzymes. The protein preparation wizard panel, which adds hydrogen atoms, removes crystallographic water molecules, and converts seleno-methionine to methionine in the proper ionization state in the physiological environment, was used to prepare the retrieved protein structure [40,41]. To achieve low 3D structures with appropriate chiralities, the identified phyto-constituents were prepared using the LigPrep panel, which generates each ligand structure’s ionization state at a physiological pH of (7.2 ± 0.2). Co-crystalized ligand was selected in the receptor grid generating window to create the glide grid file [42]. The molecular docking analysis was then completed utilizing Schrodinger’s glide, which included loading the prepared ligand’s structure and receptor grid file into Maestro’s workspace and docking the ligands using standard precision (SP) docking techniques into the receptor-binding pocket [43].

### 2.6. Statistical Analysis

All experiments were performed in triplicates, and average values were calculated using the SPSS 25.0 statistical package for Windows. Differences in means were calculated using Duncan’s multiple range tests for means with a 95% confidence interval (*p* ≤ 0.05). For anticancer activities, a significance test was carried out among the treatments by two-way ANOVA followed by Bonferroni post hoc test at *p* < 0.001.

## 3. Results

### 3.1. Chemical Composition of T. musilii Methanolic Extract

The obtained results of the tentative identification of seven tripeptides and one dipeptide with molecular weight ranging from 234.086 g/mol (Glutamyl serine) to 430 g/mol (Phenylalanyl-histidyl-glutamine). Sixteen bioactive compounds were identified by using the LC/MS technique (Table 1, Supplementary Material Figure S1) represented mainly by fatty acyls (2-4-6-8-10-dodecapentaenal, 6-9-12-15-18-Tetracosapentaynoic-acid, and 13R-hydroxy-9E-11Zoctadecadienoic), three isoprenoids (10-Hydroxyloganin, 7-epiloganin-tetraacetate, and Taxa-4(20),11(12)-dien-5 alpha-acetoxy-10beta-ol), and two flavonoids (Epicatechin-pentaacetate and dehydrorotenone). One alkaloid compound (Emetine) was also identified together with a polyphenolic aldehyde (Gossypol), isoflavones (Irigenin and Dibenzyl Ether), and a diterpene glycoside compound (4-Ketoretinoic acid glucuronide).

**Figure 1:**
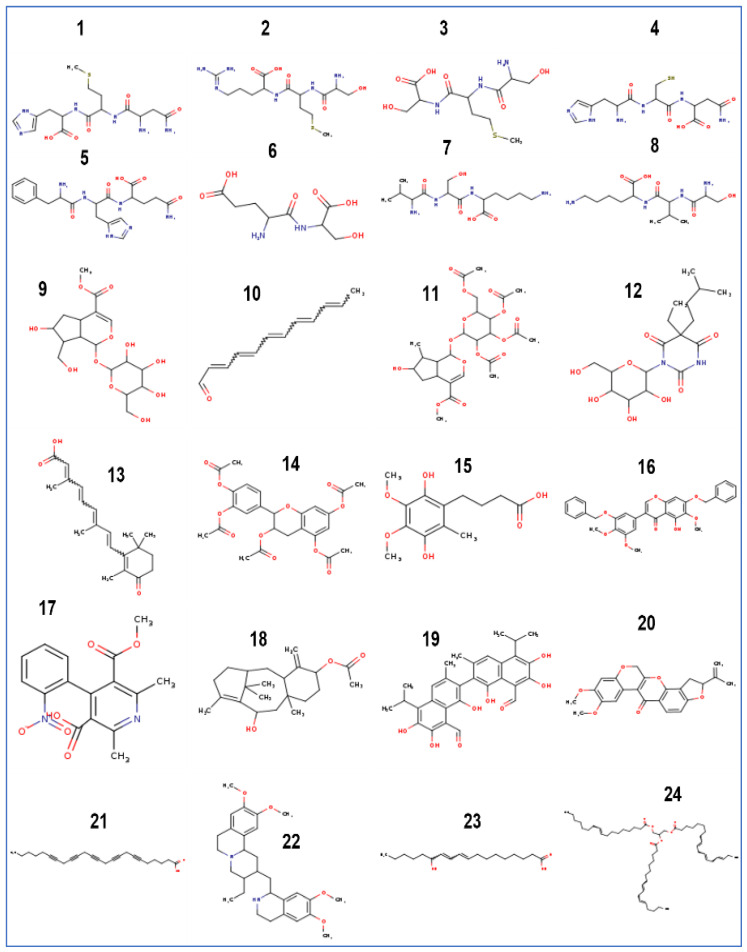
Chemical structure of the identified compounds in *T. musilii* methanolic extract. Name of the compounds are the same listed in Table 1.

The chemical structures of the identified compounds are shown in Figure 1.

### 3.2. Antimicrobial Activities of T. musilii Methanolic Extract

Table 2 summarizes the mean diameter of growth inhibition zone (mGIZ) obtained on agar media around the disc impregnated with the *T. musilii* extract (2 mg/disc), MIC, and MBC/MFC values. The results show that the tested extract was particularly active against *Candida* species with mGIZ ranging from 18.66 ± 1.52 mm for *Candida* sp. to 29.33 ± 1.15 mm (*C. albicans* ATCC 10231). While for the bacterial strains, the mean diameter of growth inhibition zone ranged from 12.33 ± 0.57 mm (*E. cloacae*) to 17.33 ± 0.57 mm (*E. coli* ATCC 35218). Moreover, the *T. musilii* extract exhibited bactericidal activity against *E. cloacae*, *S. aureus* MDR, *P. mirabilis* ATCC 29245, and *P. aeruginosa* ATCC 27,853 (MBC/MIC ratio ≤4). While the MBC/MIC ratio was higher than four for *K. pneumoniae* and *E. coli* ATCC 35218, highlighting a bacteriostatic activity against these two gram-negative bacteria. Furthermore, MIC values for yeast and molds ranged from 0.781 mg/mL to 3.125 g/mL, and MFC values ranged from 1.562 mg/mL to 25 mg/mL. The two *Aspergillus* strains were resistant to the tested extract, with the lowest mean of growth inhibition zone at about 6.00 ± 0 mm.

### 3.3. Phytochemistry and Antioxidant Activities of T. musilii Methanolic Extract

The phytochemical analysis revealed that, for *T. musilii* methanolic extract, the total flavonoid content was about 0.278 ± 0.019 mg of quercetin equivalent per gram of dry extract. Similarly, the total tannin content was evaluated at 0.084 ± 0.002 mg of tannic acid equivalent per gram of dry extract and total phenol content at 0.420 ± 0.004 mg of gallic acid equivalent per gram of dry extract. The tested extract was able to scavenge different stable free radicals with different degrees as compared to standard molecules: DPPH^•^ free radicals (IC_50_ = 0.077 ± 0.0015 mg/mL), ABTS^•+^ radicals (IC_50_ = 0.040 ± 0.0011 mg/mL), β-carotene radicals (IC_50_ = 0.278 ± 0.0012 mg/mL), and FRAP radicals (IC_50_ = 0.106 ± 0.007 mg/mL). These data are summarized in Table 3.

With the DPPH method, about a 3.34-fold difference and a 3.5-fold difference was established between *T. musilii* methanolic extract and BHT and ascorbic acid (AA), respectively. While with the ABTS method, a 2.22-fold difference and a 1.9-fold difference was noticed as compared to the same standard molecules. For the β-carotene assay, a significant difference (*p* < 0.005) between *T. musilii* and the standard molecules was established (a 6.83-fold difference with BHT and a 16.88-fold difference with AA). While only a 1.17-fold to 2.12-fold difference was established between T. *musilii* methanolic extract and BHT and AA for the FRAP assay.

### 3.4. Antivirulence Activity of T. musilii Methanolic Extract

The motility in the *P. aeruginosa* PAO1 strain is under the control of the quorum sensing system. We tested the effect of *T. musilii* methanolic extract to interfere with the motility (both swarming- and swimming-type) at 50, 75, and 100 µg/mL. The results (Table 4) showed that the swarming motility on Lauria–Bertani 0.3% agar was inhibited by (14.29 ± 1.00)% at 50 µg/mL and by (39.73 ± 1.50)% at 100 µg/mL. Similarly, the swimming motility was inhibited by (15.11 ± 0.50)% at 75 µg/mL and by (25.18 ± 1.00)% at 100 µg/mL.

The ability of *T. musilii* to inhibit the formation of biofilm of *S. aureus* ATCC 25923, *E. faecalis* ATCC 19433, *E. coli* ATCC 25922, *P. aeruginosa* ATCC 27853, *S.* Typhimurium ATCC 14028, and *C. albicans* ATCC 10,239 was tested by crystal violet staining. The results summarized in Table 5 showed that sub-MIC values were sufficient to inhibit the formation of a biofilm on polystyrene 96-well plates (21.67 ± 1.58)% for *S. aureus* ATCC 25,923 (MIC/2 = 2.5 mg/mL), (17.24 ± 1.37)% for *L. monocytogenes* ATCC 7644 (MIC/2 = 5 mg/mL), (25.41 ± 2.24)% for *E. coli* ATCC 25,922 (MIC/2 = 2.5 mg/mL), and (36.59 ± 2.84)% for *S. typhi* ATCC 14,028 (MIC/2 = 5 mg/mL). At 5 mg/mL (MIC value for *S. aureus* ATCC 25,923 and *E. coli* ATCC 25922), the biofilm formation was reduced by (42.29 ± 2.39)% and (41.96 ± 3.42)%, respectively. At 10 mg/mL (MIC value for *S.* Typhimurium ATCC 14,028 and *L. monocytogenes* ATCC 7644), the percentage of biofilm inhibition was about (53.96 ± 4.21)% and (49.54 ± 4.50)%, respectively. Interestingly, the tested extract was not able to inhibit the biofilm formation at MIC and >MIC concentrations for *P. aeruginosa* ATCC 27,853 and *C. albicans* ATCC 10239.

### 3.5. Anticancer Activity of T. musilii Methanolic Extract

**Figure 2:**
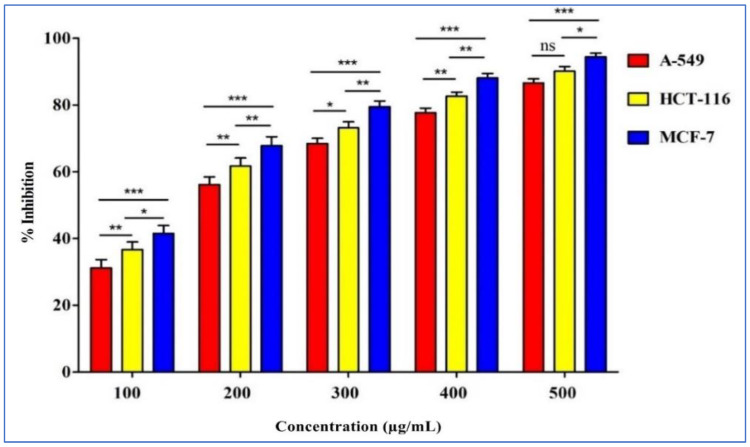
Effect of the *T. musilii* extract on breast (MCF-7), lung (A549), and colon (HCT-116) cancer cell lines according to concentration variation (100 μg/mL to 500 μg/mL) determined by MTT assay. Error bars indicate SEM (standard error of the mean) of three independent experiments. Error bars indicate SD (± standard deviation) of three independent experiments. Significance: ns, *p* > 0.01, * *p* < 0.01, ** *p* < 0.001, *** *p* < 0.0001.

The results (Figure 2) of the anticancer activities of *T. musilii* methanolic extract tested against three cell lines (MCF-7, A549, and HCT-116) showed promising results with an increasing percentage of cell viability inhibition depending on the concentration used.

In fact, the highest percentage values were obtained at 500 μg/mL against breast cancer cells with 94.38 ± 0.041%, 90.10 ± 0.126% against colon cancer cell lines (HCT-116), and 86.59 ± 0.147% against lung cancer cell lines (A549).

### 3.6. Computational Study

#### 3.6.1. ADME Analysis

**Figure 3:**
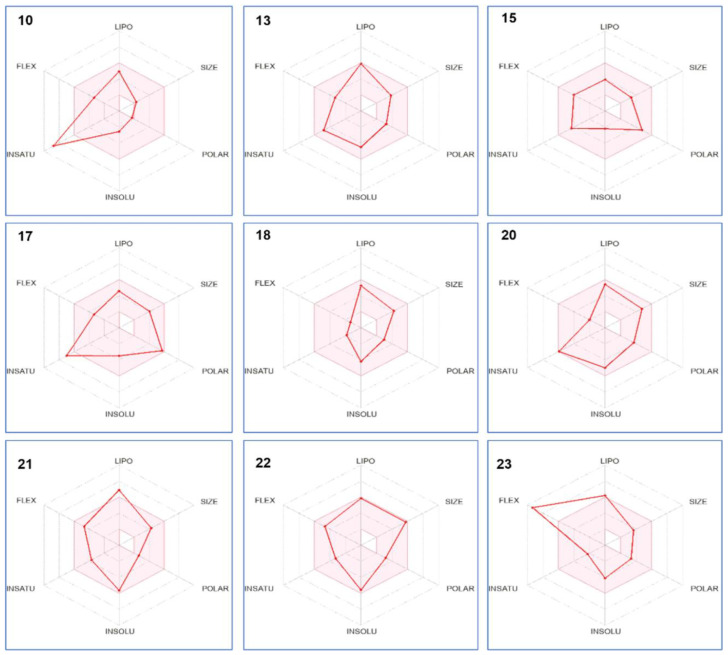
Bioavailability radar of identified compounds based on physicochemical indices ideal for oral bioavailability. LIPO—lipophilicity: −0.7 < XLOGP3 < þ 5; SIZE—molecular size: 150 g/mol < mol. wt. < 500 g/mol; POLAR—polarity: 20 Å2 < TPSA <130 Å2; INSOLU—insolubility: 0 < Log S (ESOL) < 6; INSATU—insaturation: 0.25 < Fraction Csp3 < 1; FLEX—flexibility: 0 < number of rotatable bonds < 9. The colored zone is the suitable physicochemical space for oral bioavailability.

The ADME (absorption, distribution, metabolism, and excretion) properties of all identified compounds (24 phyto-compounds) were studied. Table 6 summarizes the ADME properties of the most interesting molecules (10, 13, 15, 17, 18, 20, 21, 22, and 23). The predicted results showed that the selected compounds did not violate Lipinski’s rule and possessed a good bioavailability score ranging from 0.55 to 0.85. The highest bioavailability score (0.85) was recorded for the two compounds 4-Ketoretinoic acid glucuronide and 6-9-12-15-18-Tetracosapentaynoic-acid. In addition, all selected compounds exhibited good topological polar surface area values (TPSAs) lower than 125 Å2, suggesting that they are expected to be orally absorbed. The consensus Log Po/w value was acceptable for all selected bioactive compounds varying from 1.62 (2,5-dihydroxy-3,4-dimethoxy-6-methyl- Benzene butanoic acid) to 5.91 (6-9-12-15-18-Tetracosapentaynoic-acid). For the pharmacokinetics properties, most selected compounds had high gastrointestinal absorption. In addition, most compounds were blood–brain barrier (BBB) permeant. Only one selected molecule was a P-gp substrate (Emetine). Furthermore, the selected molecules could inhibit one or more cytochrome P450 isoenzymes. Interestingly, compound 20 (Dehydrorotenone) was able to inhibit four cytochrome P450 isoenzymes (CYP 1A2, CYP2C19, CYP2C9, and CYP3A4). All selected compounds exhibited negative Log Kp values (skin permeability) ranging from −3.79 to −6.99, highlighting their suitability as good compounds to be delivered transdermally. Figure 3 represents the bioavailability radar showing the drug-likeness behavior of all selected compounds. The results showed that most compounds were within the pink area of the polygon.

#### 3.6.2. Molecular Docking Analysis

The binding affinities and molecular interactions of various receptors involved in these biological activities were evaluated in order to obtain a better understanding of the mechanistic effects behind the biological impacts of the identified phyto-constituents, as shown in Supplementary Material Table S1. All the identified phyto-constituents displayed negative binding energies (ranging from −0.5 to −10 kcal/mol) with the different targeted receptors, among them a complex with 1JIJ, 2XCT, and 1JNX, and the short peptide Asn-Met-His scored the highest of all the receptors examined.

On the other hand, His-Cys-Asn (−5.022 kcal/mol) and Phe-His-Gln (−10.062 kcal/mol) had promising docking scores on 1HD2 and 4UYA receptors, respectively. Less affinity was observed for the identified phyto-compounds in the fungal target 2QZW, and the isoprenoid 10-Hydroxyloganin (−5.008 kcasl/mol) had the highest docking score ligand among them. In the human mitotic kinesin Eg5 receptor BBG, 4-Ketoretinoic acid glucuronide had the highest docking score of −8.794 kcal/mol, which is better than the co-crystalized inhibitor. Maestro’s ligand interaction tool was used to generate the 2D graphical representations of the ligand–protein interactions shown in Supplementary Material Figure S2.

## 4. Discussion

In this study, we report the phytochemical composition and biological activities of *T. musilii* methanolic extract using both experimental and computational work. The yield of extraction was about 7.066 ± 0.189%. We also reported the tentative identification of 16 molecules, and 7-Epiloganin tetraacetate, dehydrorotenone, 4-Ketoretinoic acid glucuronide, and 6-9-12-15-18 Tetracosapentaynoic acid were the dominant identified compounds (Table 1, Supplementary Material S1). There have been no reports on the chemical composition of *T. musilii* methanolic extract, and only one scientific work reporting the chemical composition and biological activities of *T. musilii* essential oil is available in the literature. In fact, Mseddi et al. [16] reported the identification of 17 compounds in *T. musilii* essential oil belonging mainly to oxygenated monoterpenes (87.01 ± 0.279%) that were dominated by thymol (67.697 ± 0.938%), thymyl acetate (12.993 ± 0.221%), *p*-cymene (4.617 ± 0.119%), carvacrol (3.417 ± 0.105%), and *γ*-terpinene (2.633 ± 0.072%).

It is well documented that plant species belonging to *Thymus* genus are a rich source of phenolic compounds, flavonoids, tannins, sterols, alkaloids, saponins, and polysaccharides [44,45,46,47]. In fact, Sonmezdag and colleagues [48] demonstrated that *T. serpyllum* hydroalcoholic extract studied by the high-performance liquid chromatography/electrospray ionization tandem mass spectrometry (LC-ESI-MS/MS) technique contains ten flavan-3-ols and eight phenolic acids. The dominant compounds (mg/g dry herb) were Luteolin 7-O-glucoside (51.84), luteolin (48.04), rosmarinic acid (21.72), Methyl kaempferol O-rutinoside (17.42), and Kaempferol O-glucuronide (15.21). More recently, Patil et al. [49] demonstrated that *T. vulgaris* methanolic extract is a rich source of phenolic compounds (quinic acid, rosmarinic acid, caffeic acid, p-coumaric acid, p-hydroxybenzoic acid, gentisic acid, syringic acid, and ferulic acid) and flavonoids (apigenin, luteolin, cirsimaritin, xanthomicrol, thymonin, sideritoflavone, 7-methoxyluteolin, gardenin B, salvigenin, thymusin, and 8-methoxycirsilineol) [49].

Moreover, the identified compounds 10-Hydroxyloganin and 7-epiloganin-tetraacetate (isoprenoids) were previously described in the methanolic extract from *Teucrium polium* L. plant species [24,50]. Moreover, 10-Hydroxyloganin was also reported in two perennial herbaceous plant species, namely, *Galium verum* L. and *G. mollugo* L. [51]. In addition, 7-epiloganin-tetraacetate, dehydrorotenone, and 13R-hydroxy 9E,11 Z octadecadienoic acid were identified in the methanolic extracts of the root, stem, and leaf of *Oroxylum indicum* through HR-LCMS analysis [52]. The compound 2-4-6-8-10-dodecapentaenal was identified by LC/MS technique in propolis extracts with high antimicrobial activities [53]. Epicatechin pentaacetate was reported in the ethyl acetate fraction from *Paullinia cupana* [54]. Emetine, a potent alkaloid, was also reported from *Psychotria ipecacuanha* [44,45]. Idebenone metabolites (Benzenebutanoic acid and 2,5-dihydroxy-3,4-dimethoxy-6-methyl-) were previously reported in the methanolic and ethanolic extracts of *Monotheca buxifolia* [55] and from the butanolic fraction of *Delphinium brunonianum* together [56]. Gossypol, firstly isolated in 1889 from the seeds, roots, and stems of *Gossypium* members [57,58], was also reported in our study in the methanolic extract of *T. musilii*. Similarly, tripeptides and dipeptides were successfully identified by using the HR-LCMS technique in the organic extracts from *T. polium* [24], *D. brunonianum* [55], and *A. subhirsutum* [59,60].

*T. musilii* extract was screened for its antimicrobial activities against six bacterial, four yeast, and two mold strains. The results showed high activity against *C. albicans*, *C. neoformans*, *C. vaginalis*, *Candida* sp., *E. coli*, *K. pneumoniae*, *S. aureus* MDR, *P. mirabilis*, and *P. aeruginosa* with the mean diameter of growth inhibition zone higher than 12 mm and low MIC values (0.781 to 12.5 mg/mL) and MBC (1.562 to 100 mg/mL) values. Only one scientific report has reported the antimicrobial activity of the essential oil obtained by hydrodistillation from the same plant species [16]. These authors reported that the thymol-rich essential oil of *T. musilii* was active against the same bacterial and fungal strains tested in the present work. In fact, all tested strains were sensitive to *T. musilii* essential oil with the mean diameter of growth inhibition zone for bacteria ranging from 21.33 ± 1.52 mm (*P. mirabilis*) to 36.33 ± 1.15 mm (*K. pneumoniae*). The same results were recorded with *Candida* and *Cryptococcus* strains (34.00 ± 1.00 to 37.33 ± 1.15 mm) [16]. Interestingly, the two *Aspergillus* species were resistant to *T. musilii* methanolic extract (mGIZ = 6.00 ± 0.00 mm) and highly sensitive to *T. musilii* essential oil [16]. It is well known that hydroalcoholic extracts from members of the *Thymus* genus are active against many bacterial and fungal species [61,62,63,64]. In fact, it has been demonstrated that a methanolic extract from *T. daenensis* was active against *S. aureus* ATCC 25923, *S. aureus* ATCC 29737, *S. aureus* ATCC 6538, methicillin-resistant *S. aureus* ATCC 33591, *E. faecalis* ATCC 29212, vancomycin-resistant *E. faecium*, *Micrococcus luteus* ATCC 9341, and *Streptococcus pyogenes* ATCC 8668 [65]. Similarly, a methanolic extract from T. leucotrichius was active against *E. coli* H7:O157, *B. cereus* FM 19, *B. megaterium* DSM 32, *Kluvyeromyces fragilis*, *C. albicans* ATCC 10231, and *Fusarium proliferatum* NRRL 26,517 [62]. In addition, a hydroalcoholic extract from *T. vulgaris*, *T. vulgaris, T. serpyllum, T. pulegioides, T. glabrescens, T. marschallianus, T. seravschanicus, T. sipyleus, T. algeriensis*, and *T. capitatus* was active against a large panel of bacteria, fungi, and molds [66,67,68,69,70,71,72,73,74,75].

Antioxidant activity is usually measured by quantifying the ability of antioxidant compounds (electron donors/reducing agents) to quench free radicals. Plant phyto-compounds are involved in different chemical pathways to quench reactive oxygen species and free radicals (the transfer of hydrogen and/or single electron transfer) and the reduction in metal ions [76,77]. In this study, we investigated the antioxidant potential of *T. musilii* methanolic extract by using four different assays to obtain information about the full antioxidant capacity of the tested extract. The results revealed that *T. musilii* methanolic extract was able to scavenge all tested free radicals at low concentrations as compared to *T. musilii* essential oil (DPPH^•^ IC_50_ 0.049 ± 1 × 10^−4^ mg/mL, ABTS^•+^ IC_50_ 5.6 × 10^−4^ ± 2 × 10^−5^ mg/mL, β-carotene IC_50_ = 3.20 × 10^−3^ ± 5 × 10^−4^, and FRAP IC_50_ > 1 mg/mL) [16]. The tested single, pure standard molecules (BHT and ascorbic acid) were able to scavenge DPPH^•^ and ABTS^•+^ radicals at low concentrations as compared to *T. musilii* extract. The same results were reported for the β-carotene bleaching activity and the reduction in ferric ions.

Our results are in accordance with previous works, which reported that methanolic extract from *T. leucotrichius* was able to scavenge 50% of DPPH^•^ free radicals at 43.53 µg/mL [62]. The IC_50_ of DPPH^•^ radical quenching was about 99 ± 1.06 μg/mL for *T. vulgaris* (hexanoic extract) [78], 6.00 ± 0.01 μg/mL for aqueous *T. capitatus* extract [75], 7.00 ± 0.02 μg/mL for *T. algeriensis* aqueous extract [75], 42.76 ± 1.40 μg/mL for *T. numidicus* butanoic extract [61], 24.23 ± 0.29 μg/mL for *T. seravschanicus* (ethanol–water extract) [62], and 24.23 ± 0.29 μg/mL for *T. marshallianus* (ethanol–water extract) [71]. The IC_50_ needed for the reduction in ferric ions obtained with *T. musilii* (106 ± 0.007 μg/mL) was lower compared to that obtained with *T. capitatus* and *T. algeriensis* aqueous extracts (120 ± 1.01 μg/mL and 210 ± 2.11 μg/mL) [75]. All these differences can be attributed to the chemical composition of the different extracts from *Thymus* species and especially their richness in phenolic compounds (mainly thymol and carvacrol) acting as free radical scavengers, metal ion chelators, and inhibitors of oxidative enzymes [79].

Moreover, it has been well documented that *T. vulgaris* aqueous extract containing polysaccharides (starch, homogalacturonan, and rhamnogalacturonan) was able to increase (in vitro) enzymatic antioxidant activity, such as catalase, glutathione, glutathione-S transferase, and superoxide dismutase [80]. Using Pearson’s correlation coefficient (Table 7), the statistical analysis revealed positive and significant correlation (*p* < 0.01) between TPC and TFC (r = −1.000) and TFC and TTC (r = −1.000).

The negative Pearson’s correlation coefficients highlighted that TPC and TTC are the main classes that may explain the antioxidant profile of *T. musilii* methanolic extract. Similarly, there was a positive and significant correlation (*p* < 0.01) between TPC and both DPPH (r = −1.000) and β-carotene (r = −1.000) assays. Similarly, a strong correlation was noticed between TTC and all the four used assays. For TFC, a strong positive correlation was established with DPPH (r = −1.000; *p* < 0.01), β-carotene (r = −1.000; *p* < 0.01), ABTS (r=-0.996; *p* < 0.05), and FRAP (r = −0.995; *p* < 0.05) assays. A correlation between the tested antioxidant assays with TFC were positively high (0.995 < r < 1.000), indicating that *T. musilii* methanolic extract possesses comparable activities in all four assays. In fact, it has been well documented that TPC, TFC, and TTC found in the plant kingdom are responsible for their antioxidant activities due to their redox properties [81,82,83,84,85,86].

In addition, *T. musilii* methanolic extract was able to reduce the motility of the *P. aeruginosa* PAO1 strain at low concentrations with a percentage of inhibition ranging from 14.29 ± 1.00 (at 50 µg/mL) to 39.73 ± 1.50 (at 100 µg/mL) for swarming activity and from 15.11 ± 0.50 (at 75 µg/mL) to 25.18 ± 1.00 (at 100 µg/mL) for swimming activity. Similarly, *T. musilii* methanolic extract inhibited the biofilm production of *S. aureus* ATTC 25923, *L. monocytogenes* ATCC 7644, *E. coli* ATCC 25922, and *S.* Typhimurium ATCC 14,028 at sub-MIC values with different degrees. Previous reports have demonstrated that thyme extract (*Thymus* sp.) decreased the production of violacein by 41% in *Chromobacterium violaceum* CV026 and swarming motility in *P. aeruginosa* PAO1 and *E. coli* O157:H7 by 48% and 17%, respectively [87]. In addition, Mohsenipour et al. [68] demonstrated that both ethanolic (80%) and methanolic (96%) extracts from *T. vulgaris* were able to inhibit the biofilm formation of *S. aureus*, *B. cereus*, *S. pneumoniae*, *P. aeruginosa*, *E. coli*, and *K. pneumoniae* in a concentration-dependent manner. At 20 mg/mL, the highest inhibition of biofilm formation was observed against *S. pneumoniae* (88.51%) and the lowest inhibition was observed for the biofilm formation of *E. coli* (56.83%). Similarly, the same authors [68] showed that both ethanolic and methanolic extracts from *T. vulgaris* were able to disrupt mature biofilm, and *S. pneumoniae* was the most sensitive structure (65.82%) with a mean value of destruction of about 43.15% for all tested bacteria. In addition, thyme (*T. vulgaris*) ethanolic extract was able to inhibit the biofilm formation of *Campylobacter jejuni* on an abiotic surface (polystyrene) up to 35% at concentrations of 50–200 μg/mL [88]. More recently, Mulugeta and colleagues showed that *T. vulgaris* crude extract obtained using the Soxhlet method attenuated the production of pyocyanin, LasA, violacein, and swarming motility and exhibited antibiofilm activities towards *E. coli*, *S. aureus*, *P. aeruginosa*, and *S. typhimurium* foodborne pathogenic bacteria [89].

We also tested the anticancer activity of *T. musilii* methanolic extract on three different cell lines (MCF-7, A549, and HCT-116). The results showed an IC_50_ value about 153.54 μg/mL, 107.69 μg/mL, and 194.70 μg/mL, respectively, for HCT-116, MCF-7, and A-549 cell lines. These results coincide with previous works highlighting the promising anticancer activities of organic extracts from *Thymus* members [90,91,92]. In an extensive literature review paper, Alfonso and colleagues [93] reported the anticancer activities of various species belonging to the *Thymus* genus, including methanolic extracts from *T. algeriensis* and *T. serpyllum*, ethanol/water and aqueous extracts from *T. vulgaris*, *T. sygis* subsp. *sygis*, *T. caramanicus, T. carnosus, T. citriodorus*, *T. mastichina*, *T. pulegioides*, aqueous extract from *T. serpyllum*, *T. vulgaris*, and *T. satureioides*, and, finally, from a methanol 70% extract of *T. schimperi*. These results can be correlated to the presence of various bioactive compounds in the methanolic extract tested (isoprenoids, flavonoids, alkaloids, and terpenoids). In fact, Emetine, a potent alkaloid, is known to possess antiviral activities [60,94,95]. It is interesting to mention that Emetine was described to possess anticancer potential on many malignant cell lines including leukemia cell lines (U937 and CEM/ADR5000), lung cell lines (A549-S), human promyelocytic leukemia cells (CCRFCEM), T cell leukemia (Jurkat T cells), rat hepatocytes, and human T cell lymphoblast-like cells [96,97,98,99]. In addition, the polyphenolic compound (Gossypol) has been described to possess antifungal [100] and anticancer activities against prostate [101], colon [102], pancreas [103], and breast cancer [104].

A computational approach using ADME analysis of the pharmacokinetic, physiochemical, and drug-likeness properties of the identified molecules in *T. musilii* methanolic extract revealed that eight molecules were depicted to have good bioavailability score, topological polar surface area values (TPSAs), consensus Log Po/w values, and high gastrointestinal properties. Moreover, in silico mathematical analysis showed that for TyrRS *S. aureus* (1JIJ), the docking scores of phyto-constituents ranged from −2.234 to −7.566 kcal/mol, where three ligands showed docking scores comparable to those of the co-crystalized ligand (−7.973 kcal/mol). In this target, Asn-Met-His had the best docking score, which was −7.566. The polar group of Asn-Met-His and the reference compound (co-crystalized ligand) interacted with Asp195(1.90), Asp40(2.14), Asp177(2.59), and Asp80(2.30) at the binding site via a hydrogen bond [105]. In addition, salt bridge interactions were observed between Asn-Met-His and Asp80(3.14), lys84(4.78), and Asp195 (2.99). The results of the molecular docking at the active site *S. aureus* DNA gyrase (2XCT) revealed that most of the docked ligands had docking scores better than the reference Ciprofloxacin (−8.521 kcal/mol) [106]. Among the screened library short peptides, namely, Asn-Met-His, Ser-Met-Arg, Ser-Met-Ser, His-Cys-Asn, Phe-His-Gln, Val-Ser-Lys, and Ser-Val-Lys, these displayed a better docking score than the reference drug. The binding interaction of Asn-Met-His showed that it interacted with the Mn+2 ion through a salt bridge, which greatly enhanced rates of enzyme-mediated DNA breakage, as reported in earlier studies. Furthermore, the Asn-Met-His polar group and nucleotide bases (DG G9 and DT G10) exhibited hydrogen bonding, π–cation interactions (DA H13 and DC H12), and hydrophobic π–π interactions (DC H13). In this complex, the protonated amino terminal shows the hydrogen as well as the salt bridge bond with the charged negative amino acid Asp437. In human peroxiredoxin 5 (1HD2), the highest docking score was shown by the co-crystalized or reference ligand (−7.245 kcal/mol). However, significant binding affinities were also observed with peptides His-Cys-Asn, Ser-Val-Lys, Asn-Met-His, and Phe-His-Gln, with docking values ranging from −5.022 to −4.780 kcal/mol. The human peroxiredoxin family contains one cysteine residue, Cys47, which is conserved in all other peroxiredoxins and was directly linked to peroxide catalysis. Cys47, a conserved cysteine residue, is found within a minor cavity at the N-terminus of the kinked helix α2 [106]. This active pocket contains conserved amino acid residues, including Thr44, Gly46, Cys47, and Arg127, which are important in docked compound recognition through hydrogen bonding and hydrophobic interactions.

A detailed examination of the docking poses of the screened phyto-compounds revealed the presence of a hydrogen bond with Cys47 with the peptides His-Cys-Asn, Ser-Val-Lys, Asn-Met-His, and Phe-His-Gln, as well as the antioxidant reference ligand Benzoic Acid, indicating the antioxidant potential of the peptide. The receptor 2QZW is an aspartic proteinase (Sap) 1 secreted by *C. albicans*, which reportedly plays a key role in superficial *Candida* infections. Sap 1 is among the most crucial virulent factors generated by *C. albicans* cells since these hydrolytic enzymes engage in a variety of fungal physiological processes as well as other aspects of fungal–host interactions [107]. For these reasons, Saps clearly hold promise as new potential drug targets. A docking study on this target showed that the isoprenoid 10-Hydroxyloganin (−5.008 kcal/mol) had the highest docking score ligand among the identified phyto-compounds. The binding interaction showed that it formed five hydrogen bonds with active site residues Gly102(1.77), Lys24(2.30), Thr19(1.98), and Asp17(2.05 and 2.00). In this target, pepstatin (co-crystalized ligand) interacts with Asp86, Glu193, Arg195, and Asp37, whereas none of our identified phyto-compounds showed a comparable interaction, which is the primary reason for the lower affinity towards the *C. albicans* target Sap 1.

To evaluate the affinity of identified phyto-compounds for cancer targets, a docking investigation on the MLK4 kinase domain (4UYA), which controls the JNK, p38, and ERK kinase signaling pathways, was performed [108]. The results of molecular docking at the active site A4UYA revealed that most of the docked ligands had docking scores better than the reference (−7.897 kcal/mol), among which Phe-His-Gln (−10.062 kcal/mol), 6-9-12-15-18-Tetracosapentaynoic-acid (−10.012 kcal/mol), and Asn-Met-His (−8.983 kcal/mol) had significant docking scores. The binding interaction of the most scored peptide Phe-His-Gln shows that at physiological pH, the deprotonated carboxylate ion makes a salt bridge with Lys265, while the protonated amine group interacts with Thr288. In this complex, a π–cationic interaction with the imidazole ring and Mg^2+^ ion in the active site of the MLK4 kinase domain was also visible. The human mitotic kinesin Eg5 receptor 4BBG is essential for the formation of the mitotic spindle. Mitotic kinesins, which play an important role in mitotic cell division, are attractive anticancer therapeutic targets [109]. In this context, the kinesin Eg5 has received a lot of interest, and there are several inhibitors at various stages of clinical studies. 4-Ketoretinoic acid glucuronide had the top scoring phyto-compound in this target, with a docking score of −8.794 kcal/mol, which is more significant than co-crystalized inhibitor 3-(((2-Aminoethyl) sulfanyl) (3-ethylphenyl) phenyl methyl) phenol (−8.684 kcal/mol). The binding mode of 4-Ketoretinoic acid glucuronide revealed that it had only one hydrogen bond with Arg221, while the co-crystalized ligand interacted with the acidic amino acids Glu212 and Glu116. Additionally, a van der Waals interaction was also observed during the binding of 4-Ketoretinoic acid glucuronide with the amino acid residues Pro137, Gly134, Asp130, Trp127, Try211, Leu214, Ala218, and Ala219.

The C-terminal BRCT region of BRCA1 is the receptor for 1JNX, which is required for DNA repair, tumor suppressor activities, and transcriptional regulation [110,111]. A docking study on this target showed that Asn-Met-His (−5.979 kcal/mol), Ser-Met-Arg (−5.308 kcal/mol), and Phe-His-Gln (−4.978 kcal/mol) had the most promising docking score among the identified phyto-compounds. In the binding mode, Asn-Met-His interacted with four hydrogen bonds with Cys1768(1.50), Lys1793(1.86), Glu1781 (1.62), and 1.21), sharing many common residues with the active site of the investigated enzyme. Overall, the biological effects of the phyto-constituents in *T. musilii* Velen., notably, the tripeptides, seem to be thermodynamically feasible.

## 5. Conclusions

In the present work, we reported for the first time the chemical composition of *T. musilii* methanolic extract by using the HR-LCMS technique. Several bioactive molecules belonging to different chemical classes (isoprenoids, flavonoids, alkaloids, terpenoids, polyphenols, and fatty acyls) with promising biological activities were reported. The tested extract was able to inhibit the growth of several gram-positive, gram-negative, and yeast strains at low concentrations. Similarly, good antioxidant activities were also recorded, and *T. musilii* was able to scavenge various free radicals at low concentrations. Similarly, *T. musilii* attenuated the production of some virulence properties in *P. aeruginosa* (swarming and swimming) and biofilm formation by pathogenic bacteria. In silico approaches showed a good ADME profile of most identified molecules in *T. musilii* methanolic extract and a high binding score to the tested target proteins. More studies are needed to highlight the mechanism of action of *T. musilii* crude extract and its bioactive components.

## Figures and Tables

**Table 1:** Tentative identification of bioactive molecules in *T. musilii* methanolic extract by HR-LCMS technique.

N°	Compounds	Chemical Class	Retention Time	Molecular Weight (g/mol)	Chemical Formula
**1**	Asn-Met-His	Tripeptides	2.923	400.1528	C_15_ H_24_ N_6_ O_5_ S
**2**	Ser-Met-Arg	Tripeptides	5.152	392.1808	C_14_ H_28_ N_6_ O_5_ S
**3**	Ser-Met-Ser	Tripeptides	5.271	323.1115	C_11_ H_21_ N_3_ O_6_ S
**4**	His-Cys-Asn	Tripeptides	5.445	372.118	C_13_ H_20_ N_6_ O_5_ S
**5**	Phe-His-Gln	Tripeptides	5.99	430.1967	C_20_ H_26_ N_6_ O_5_
**6**	Glu-Ser	Dipeptides	8.822	234.086	C_8_ H_14_ N_2_ O_6_
**7**	Val-Ser-Lys	Tripeptides	12.675	332.2103	C_14_ H_28_ N_4_ O_5_
**8**	Ser-Val-Lys	Tripeptides	13.674	332.2105	C_14_ H_28_ N_4_ O_5_
**9**	10-Hydroxyloganin	Isoprenoids	0.963	406.1437	C_17_ H_26_ O_11_
**10**	2-4-6-8-10-dodecapentaenal	Fatty acyls	1.081	174.1044	C_12_ H_14_ O
**11**	7-Epiloganin-tetraacetate	Isoprenoids	4.776	558.1982	C_25_ H_34_ O_14_
**12**	2-4-6-Pyrimidinetrione-5-ethyl-1-b-D-glucopyranosyl-5-1-methylbutyl	Polyunsaturated fatty acids	4.83	388.1865	C_17_ H_28_ N_2_ O_8_
**13**	4-Ketoretinoic acid glucuronide	Diterpene glycosides	6.218	490.2201	C_26_ H_34_ O_9_
**14**	Epicatechin pentaacetate	Flavonoids	7.875	500.1362	C_25_ H_24_ O_11_
**15**	Benzenebutanoic acid, 2,5-dihydroxy-3,4-dimethoxy-6-methyl-	Idebenone metabolites	7.913	270.108	C_13_ H_18_ O_6_
**16**	Irigenin, Dibenzyl ether	Isoflavones	7.89	540.1667	C_32_ H_28_ O_8_
**17**	Desmethyl dehydronifedipine	Vitamin B complex	8.532	330.0848	C_16_ H_14_ N_2_ O_6_
**18**	Taxa-4(20),11(12)-dien- 5alpha-acetoxy-10beta-ol	Isoprenoids	8.752	346.2467	C_22_ H_34_ O_3_
**19**	Gossypol	Polyphenolic aldehydes	9.316	518.1823	C_30_ H_30_ O_8_
**20**	Dehydrorotenone	Flavonoids	10.699	392.1235	C_23_ H_20_ O_6_
**21**	6-9-12-15-18-Tetracosapentaynoic-acid	Fatty acyls	11.857	348.2058	C_24_ H_28_ O_2_
**22**	Emetine	Alkaloids	15.853	480.3018	C_29_ H_40_ N_2_ O_4_
**23**	13R-hydroxy-9E-11Zoctadecadienoic	Fatty Acyls	15.943	296.2339	C_18_ H_32_ O_3_
**24**	1-(9Z-heptadecenoyl)-2-(9Z,12Z-heptadecadienoyl)-3-(9Z,12Z,15Z-octadecatrienoyl)-sn-glycerol	Glycerolipids	17.033	916.7577	C_60_ H_100_ O_6_

**Table 2:** Mean growth inhibition zones (mGIZ), MIC, MBC, and MFC values obtained for bacterial strains tested using disc diffusion and microdilution assays.

Code	Bacterial Strains	*T. musilii* Methanolic Extract				Ampicillin Mean ± SD (mm)
		mGIZ ± SD ^1^ (mm)	MIC ^2^	MBC ^3^	MBC/MIC Ratio	
**B_1_**	*E. coli* ATCC 35218	17.33 ± 0.57 ^cd^	6.25	100	16	7.00 ± 0.00 ^d^
**B_2_**	*P. aeruginosa* ATCC 27853	12.33 ± 0.57 ^f^	12.5	50	4	7.33 ±0.57 ^d^
**B_3_**	*Proteus mirabilis* ATCC 29245	15.00 ± 1.15 ^e^	6.25	25	4	6.33 ± 0.57 ^d^
**B_4_**	*K. pneumoniae* ATCC 27736	15.33 ± 0.57 ^e^	6.25	50	8	6.66 ± 0.57 ^d^
**B_9_**	*S. aureus* MDR (Clinical strain)	16.66 ± 0.57 ^de^	6.25	25	4	7.33 ± 0.57 ^d^
**B_10_**	*E. cloacae* (Clinical strain)	16.00 ± 0.00 ^de^	6.25	12.5	2	6.66 ± 0.57 ^d^
**Code**	**Yeasts and molds**	**mGIZ ± SD (mm)**	**MIC**	**MFC ^4^**	**MFC/MIC ratio**	**Amphotericin B Mean ± SD (mm)**
**Y_1_**	*C. albicans* ATCC 10231	29.33 ± 1.15 ^a^	3.125	25	8	22.66 ± 1.15 ^a^
**Y_2_**	*C. neoformans* ATCC 14116	27.00 ± 1.00 ^b^	0.781	1.562	2	15.33 ± 0.57 ^b^
**Y_3_**	*C. vaginalis* (Clinical strain)	29.33 ± 2.30 ^a^	3.125	25	8	6.66 ± 0.57 ^d^
**Y_4_**	*Candida* sp. (Clinical strain)	18.66 ± 1.52 ^c^	1.562	25	16	12.33 ± 0.57 ^c^
**M_1_**	*A. fumigatus* ATCC 204305	6.00 ± 0.00 ^g^	-	-	-	15.00 ± 1.00 ^b^
**M_2_**	*A. niger*	6.00 ± 0.00 ^g^	-	-	-	6.00 ± 0.00 ^d^

^1^: Inhibition zone around the discs expressed as mean of three replicates, SD: standard deviation. ^2^: Minimal inhibitory concentration. ^3^: Minimal bactericidal concentration (mg/mL). ^4^: Minimal fungicidal concentration (mg/mL). The letters (a–g) indicate a significant difference between the inhibition zones of *T. musilii* methanolic extract and standard drugs against the tested micro-organisms according to Duncan’s test (*p* < 0.05). Ampicillin and amphotericin B stock solutions (10 mg/mL) were used (10 µL/disc).

**Table 3:** Antioxidant activities of *T. musilii* methanolic extract as compared to standard molecules.

Tests	DPPH• IC_50_ (mg/mL)	ABTS•^+^ IC_50_ (mg/mL)	β-Carotene IC_50_ (mg/mL)	FRAP IC_50_ (mg/mL)
*T. musilii* methanolic extract	0.077 ± 0.0015	0.040 ± 0.011	0.287 ± 0.012	0.106 ± 0.007
BHT (Butylated hydroxytoluene)	0.023 ± 3 × 10^−4^	0.018 ± 4 × 10^−4^	0.042 ± 3.5 × 10^−3^	0.05 ± 3 × 10^−3^
Ascorbic Acid	0.022 ± 5 × 10^−4^	0.021 ± 1 × 10^−3^	0.017 ± 1 × 10^−3^	0.09 ± 7 × 10^−3^

**Table 4:** Effect of *T. musilii* methanolic extract on the motility of *P. aeruginosa* PAO1.

Test	*T. musilii* Methanolic Extract (µg/mL)		
	100	75	50
Swarming inhibition (%)	39.73 ± 1.50	23.67 ± 1.50	14.29 ± 1.00
Swimming inhibition (%)	25.18 ± 1.00	15.11 ± 0.50	(-)

(-): No activity.

**Table 5:** Percentage of biofilm inhibition by *T. musilii* methanolic extract tested against pathogenic bacteria.

Micro-Organisms Tested	Concentration Used	Percentage of Biofilm Inhibition (%)
***S. aureus*** **ATCC 25923**	**MIC = 5 mg/mL**	42.29 ± 2.39 ^b^
	**MIC/2 = 2.5 mg/mL**	21.67 ± 1.58 ^de^
***L. monocytogenes*** **ATCC 7644**	**MIC = 10 mg/mL**	49.54 ± 4.50 ^a^
	**MIC/2 = 5 mg/mL**	17.24 ± 1.37 ^e^
***E. coli*** **ATCC 25922**	**MIC = 5 mg/mL**	41.96 ± 3.42 ^b^
	**MIC/2 = 2.5 mg/mL**	25.41 ± 2.24 ^d^
	**MIC/4 = 1.25 mg/mL**	9.94 ± 0.55 ^f^
***S.*** ***typhimurium*** **ATCC 14028**	**MIC = 10 mg mL**	53.96 ± 4.21 ^a^
	**MIC/2 = 5 mg/mL**	36.59 ± 2.84 ^c^
	**MIC/4 = 2.5 mg/mL**	11.12 ± 0.95 ^f^

The means followed by the same letters are not significantly different at *p* = 0.05 based on Duncan’s multiple range test.

**Table 6:** Selected ADME properties of some identified compounds. Number and name of the compounds are the same as listed in Table 1.

Entry	Phyto-Constituents								
	10	13	15	17	18	20	21	22	23
Physicochemical, Lipophilicity, and Drug-Likeness properties									
Molecular weight	174.24	314.42	270.28	330.29	346.50	392.40	348.48	480.64	296.44
Num. heavy atoms	13	23	19	24	25	29	26	35	21
Num. arom. heavy atoms	0	0	6	12	0	16	0	12	0
Fraction Csp3	0.08	0.40	0.46	0.19	0.77	0.26	0.54	0.59	0.72
Num. rotatable bonds	5	5	6	5	2	3	7	7	14
Num. H-bond acceptors	1	3	6	7	3	6	2	6	3
Num. H-bond donors	0	1	3	1	1	0	1	1	2
Molar Refractivity	57.63	95.48	69.60	86.67	102.50	108.73	110.06	147.05	90.63
TPSA (Å^2^)	17.07	54.37	96.22	122.31	46.53	67.13	37.30	52.19	57.53
Consensus Log *P*_o/w_	3.01	4.19	1.62	1.67	4.03	3.83	5.91	4.19	4.54
Lipinskiˈs Rule	Yes	Yes	Yes	Yes	Yes	Yes	Yes	Yes	Yes
Bioavailability Score	0.55	0.85	0.56	0.56	0.55	0.55	0.85	0.55	0.85
Pharmacokinetic properties									
GI absorption	High	High	High	High	High	High	High	High	High
BBB permeant	No	Yes	No	No	Yes	Yes	Yes	Yes	Yes
P-gp substrate	No	No	No	No	No	No	No	Yes	No
CYP1A2 inhibitor	No	Yes	No	No	No	Yes	Yes	No	Yes
CYP2C19 inhibitor	No	Yes	No	No	No	Yes	No	No	No
CYP2C9 inhibitor	No	Yes	No	Yes	No	Yes	Yes	No	Yes
CYP2D6 inhibitor	No	No	No	No	No	No	No	No	Yes
CYP3A4 inhibitor	No	No	No	No	No	Yes	No	No	No
Log Kp (cm/s)	−5.15	−4.80	−6.99	−6.55	−5.77	−5.87	−3.79	−5.87	−4.31

**Table 7:** Pearson’s Correlation.

	TPC	TFC	TTC	DPPH	ABTS	β-Carotene	FRAP
TPC	1						
TFC	−1.000 **	1					
TTC	1.000 **	−1.000 **	1				
DPPH	−1.000 **	1.000 **	−1.000 **	1			
ABTS	−0.996 *	0.996 *	−0.996 *	0.996 *	1		
β-carotene	−1.000 **	1.000 **	−1.000 **	1.000 **	0.996 *	1	
FRAP	−0.995 *	0.995 *	−0.995 *	0.995 *	1.000 **	0.995 *	1

Significance: ** correlation is significant at 0.01 level; * correlation is significant at 0.05 level.

## Data Availability

All data generated or analyzed during this study are included in this article.

## Supplementary Materials

The following supporting information can be downloaded at: https://www.mdpi.com/article/10.3390/life13010062/s1. Figure S1: Chromatogram showing the dominant compounds identified by HR-LCMS technique in *T. musilii* methanolic extract. Figure S2: Binding interaction of promising compound identified in docking study with different receptor A. Asn-Met-His with *S. aureus* TyrRS (PDB ID: 1JIJ); B. Asn-Met-His with *S. aureus* DNA gyrase (PDB ID: 2XCT); C. His-Cys-Asn with Human peroxiredoxin 5 (PDB ID: 1HD2); D. 10-Hydroxyloganin with *C. albicans* Sap 1(PDB ID: 2QZW); E. Phe-His-Gln with MLK4 kinase domain (PDB ID: 4UYA); F. 4-Ketoretinoic acid glucuronide with human kinesin (PDB ID: 4BBG); G. Asn-Met-His with BRCT protein (PDB ID: 1JNX). Table S1. Result of the docking experiment performed between the selected target proteins and the identified phytocompounds in *T. musilii* methanolic extract.
