# Supplementary material for: Thymus musilii Velen. Methanolic Extract: In Vitro and In Silico Screening of Its Antimicrobial, Antioxidant, Anti-Quorum Sensing, Antibiofilm, and Anticancer Activities
Source: Life (Basel). 2022 Dec 25;13(1):62. doi: 10.3390/life13010062 (PMC9862435; doi:10.3390/life13010062)
Supplement: Supplementary file 1 [file life-13-00062-s001.zip › life-2059252-supplementary.pdf]

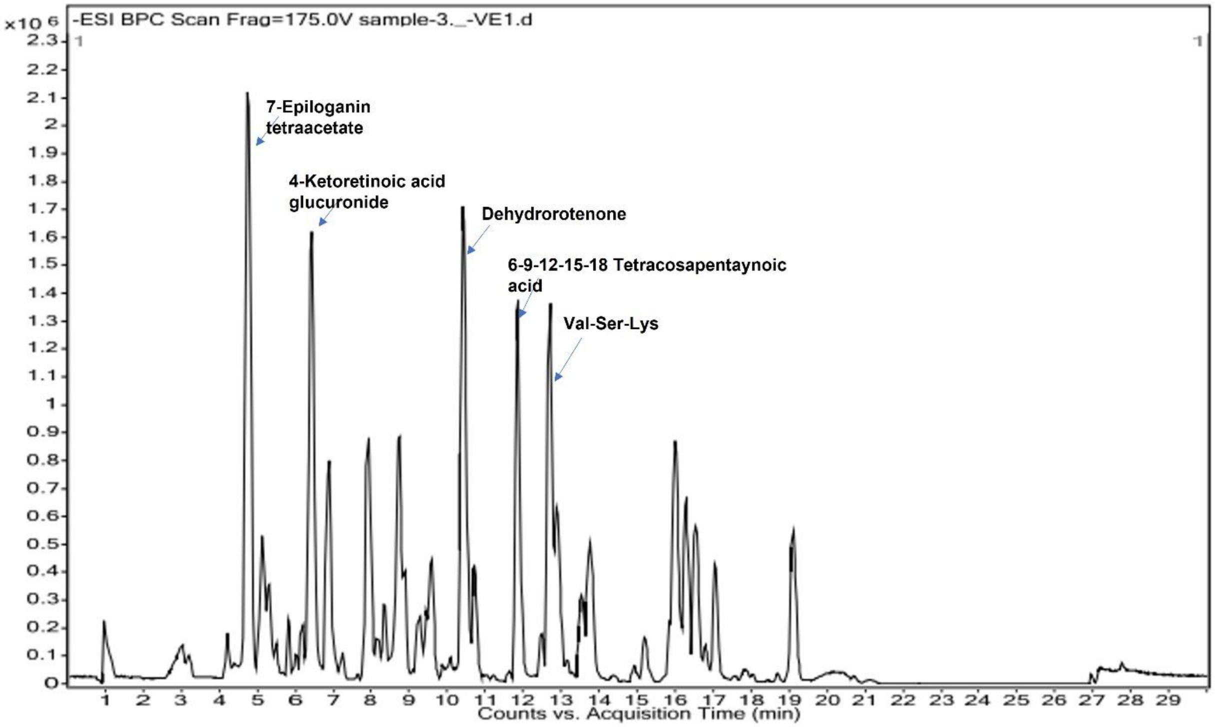

Supplementary Material Figure S1: Chromatogram showing the dominant compounds identified by HR-LCMS technique in *T. musilii* methanolic extract.

Supplementary material Table S1. Result of the docking experiment performed between the selected target proteins and the identified phytocompounds in *T. musilii* methanolic extract.

No.	Compounds	1JIJ	2XCT	1HD2	2QZW	4UYA	4BBG	1JNX
**1**	Asn-Met-His	**-7.566**	**-9.705**	-4.929	-3.031	-8.983	-6.626	**-5.379**
**2**	Ser-Met-Arg	-6.368	-9.035	-2.432	-3.569	-7.372	-5.386	-5.908
**3**	Ser-Met-Ser	-7.191	-9.132	-4.603	-4.837	-7.278	-6.594	-3.837
**4**	His-Cys-Asn	-7.383	-9.077	**-5.022**	-3.528	-8.69	-6.166	-4.64
**5**	Phe-His-Gln	-6.523	-9.252	-4.780	-4.888	**-10.062**	-6.202	-4.978
**6**	Glu-Ser	-5.66	-5.691	-1.831	-1.736	-6.174	-4.918	-2.361
**7**	Val-Ser-Lys	-7.019	-8.721	-4.32	-2.915	-8.191	-4.353	-4.595
**8**	Ser-Val-Lys	-6.088	-9.25	-5.047	-2.899	-7.73	-4.988	-4.487
**9**	10-Hydroxyloganin	-5.995	-7.902	-4.50	**-5.008**	-4.942	-6.034	-3.718
**10**	2-4-6-8-10-dodecapentaenal	-3.465	-6.75	-0.956	NA	-3.72	-2.251	-2.092
**11**	7-Epiloganin-tetraacetate	-5.66	-4.683	NA	-3.038	-6.94	-5.046	-4.172
**12**	2-4-6-Pyrimidinetrione-5-ethyl-1-b-D-glucopyranosyl-5-1-methylbutyl	-3.215	-4.621	-4.951	-2.514	-4.564	-2.315	-3.916
**13**	4-Ketoretinoic acid glucuronide	-5.41	-8.342	NA	-2.917	-8.87	**-8.794**	-3.911
**14**	Epicatechin-pentaacetate	-6.335	-7.692	-2.527	-4.065	-7.301	-4.692	-3.112
**15**	Benzenebutanoic acid, 2,5-dihydroxy-3,4-dimethoxy-6-methyl-	-4.943	-8.353	-3.83	-3.353	-9.05	-4.781	-3.38
**16**	Irigenin, Dibenzyl ether	-5.277	-5.916	NA	-4.656	-5.892	-5.728	-4.229
**17**	Desmethyl dehydronifedipine	-4.145	-5.414	-3.514	-4.452	-5.42	-5.144	-4.741
**18**	11(12)-dien- 5alpha-acetoxy-10beta-ol	-3.276	-3.666	-2.953	-2.746	-4.678	-4.674	-2.284
**19**	Gossypol	-4.439	-9.148	NA	-3.364	-9.127	-4.392	-3.262
**20**	Dehydrorotenone	-5.499	-8.15	NA	-3.36	-5.837	-5.064	-3.48
**21**	6-9-12-15-18-Tetracosapentaynoic-acid	-3.384	-4.91	NA	-2.253	-10.012	-4.077	-2.558
**22**	Emetine	-6.808	-7.477	-1.563	-2.549	-7.034	-4.812	-3.154
**23**	13R-hydroxy-9E-11Zoctadecadienoic	-2.234	-4.237	-1.073	-0.723	-4.791	-2.312	-0.544
**24**	1-(9Z-heptadecenoyl)-2-(9Z,12Z-heptadecadienoyl)-3-(9Z,12Z,15Z-octadecatrienoyl)-sn-glycerol	NA	NA	NA	-3.768	-7.379	NA	-3.446
**-**	**Reference ligand/ Co-crystal inhibitor**	**-7.973**	**-8.521**	**-7.245**	**-7.851**	**-7.897**	**-8.684**	**-**

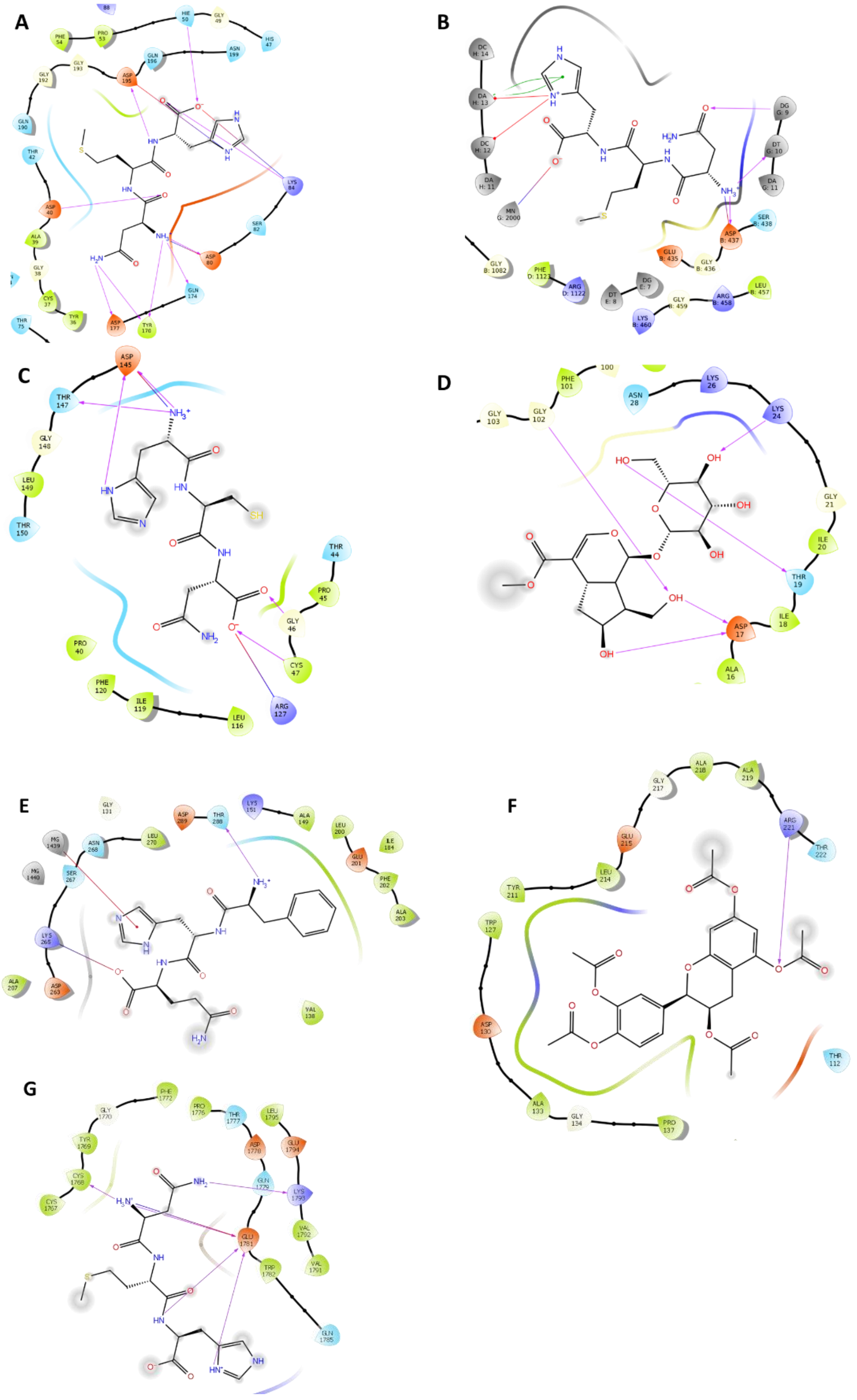

Supplementary material Figure S2. Binding interaction of promising compound identified in docking study with different receptor **A**. Asn-Met-His with *S. aureus* TyrRS (PDB ID: 1JIJ); **B**. Asn-Met-His with *S. aureus* DNA gyrase (PDB ID: 2XCT); **C**. His-Cys-Asn with Human peroxiredoxin 5 (PDB ID: 1HD2); **D**. 10-Hydroxyloganin with *C. albicans* Sap 1(PDB ID: 2QZW); **E**. Phe-His-Gln with MLK4 kinase domain (PDB ID: 4UYA); **F**. 4-Ketoretinoic acid glucuronide with human kinesin (PDB ID: 4BBG); **G**. Asn-Met-His with BRCT protein (PDB ID: 1JNX).
